# *Leishmania tarentolae* molecular signatures in a 300 hundred-years-old human Brazilian mummy

**DOI:** 10.1186/s13071-015-0666-z

**Published:** 2015-02-04

**Authors:** Shênia PC Novo, Daniela Leles, Raffaella Bianucci, Adauto Araujo

**Affiliations:** Departamento de Endemias Samuel Pessoa, Escola Nacional de Saúde Pública Sergio Arouca, Fundação Oswaldo Cruz, Fiocruz, rua Leopoldo Bulhões, 1480, Térreo, Manguinhos, 21041-210 Rio de Janeiro Brasil; Departamento de Microbiologia e Parasitologia, Instituto Biomédico, Universidade Federal Fluminense, Laboratório de Biologia Molecular de Parasitos, Rua Professor Hernani Melo 101, São domingos, Niterói, Rio de Janeiro 24210-130 Brazil; Department of Public Health and Paediatric Sciences, Laboratory of Physical Anthropology, University of Turin, Corso Galileo Galilei, 22, 10126 Turin, Italy; Center for Ecological and Evolutionary Synthesis (CEES), Department of Biosciences, University of Oslo, P.O. Box 1066, Blindern, NO-0316 Oslo Norway; Anthropologie bioculturelle, Droit, Ethique et Santé, Aix-Marseille Université, 15, boulevard Pierre Dramard, Faculté de Médecine-Nord, Cedex 15, 13344 Marseille, France

**Keywords:** *Leishmania tarentolae*, Lizards, Minas Gerais, Ancient DNA, New World

## Abstract

**Background:**

*L. tarentolae*, the lizard-infecting species of Old World geckos, has been classified as non-pathogenic to man. While it has been demonstrated that *L. tarentolae* is capable of infecting human phagocytic cells and to differentiate into amastigote-like forms, there is no clear evidence for its efficient replication within macrophages. Here we provide first evidence for *L. tarentolae* ancient DNA sequences from bone marrow and intestines of a 300yo adult male.

**Methods:**

We identified molecular signatures of *Leishmania tarentolae,* the lizard-infecting species of Old World geckos, in hard and soft tissue biopsies from a Brazilian mummy (A74) uncovered in Itacambira (Brazil) and dating to the Colonial Period (end of 18th/beginning of the 19th century).

**Results:**

Our results imply that efficient replication of the parasite occurred within human macrophage and to lead to a systemic spread and visceralization in this individual. The ancient sequences show a 100% similarity with those of isolated *L. tarentolae* parasites grown on artificial nutrient media and a 99% similarity with two modern sequences isolated from reptiles.

**Conclusions:**

*De facto*, our findings re-open the debate about the potential survival of ancient *L. tarentolae* strain within human macrophage and its ability to spread systemically. They also raise ecological issues since it is unknown whether this parasite circulates in the reptilian reservoir in modern day Brazil or not. Investigations on fossil fauna and arthropods are needed to shed light on the interactions between saurian *Leishmania* and lizards in Brazil’s remote and recent past.

## Background

Human leishmaniases are responsible for heavy disease burden in modern populations. Three hundred million people are at risk with an annual incidence of 2 million and an overall prevalence estimated at 12 million people worldwide [1].

Different forms of the disease can be encountered in various areas of the globe and range from self-healing cutaneous lesions (CL) to mucocutaneous (MCL) and severe health-threatening visceral infections (VL) [2]. Up till the present day, at least 21 species belonging to the genus *Leishmania* have been classified as human pathogens [3,4] but the global number of species responsible for human disease still remains controversial [5,6]. Along with known reservoirs of leishmanias in mammals, there is a group of reptiles, mainly lizards, which harbors *Leishmania* infections. Their role in spreading leishmaniasis is far from being completely understood [7,8]. Inclusion of these species in genus *Leishmania* has been controversial especially because the presence of their intracellular amastigote forms in the host has never been well established [9,10]*.*

The reptilian species were, therefore, classified in a separate genus named *Sauroleishmania* [5,6,11]. The molecular data, whole-genome sequencing data included, showed that the genus *Leishmania* is monophyletic group with three distinct subgenera *Leishmania* (*Leishmania*), *Leishmania* (*Viannia*), and *Leishmania* (*Sauroleishmania*) [3,12].

On this ground, subsequent molecular phylogenetic studies have provided robust evidence for the hypothesis that *Sauroleishmania* species have evolved from the mammalian *Leishmania* [13].

Subgenus *Sauroleishmania* includes the species *Leishmania tarentolae*. Initially isolated in *Tarentolae mauritanica* [14], *L. tarentolae* was also found in gecko species (*Gekkonidae*) from other parts of Africa and from the Mediterranean basin.

Sand flies belonging to the genus *Sergentomyia,* which feed on reptiles, are the known vectors of *L. tarentolae* in the Old World [15-22].

Once ingested, the reptilian blood is stored in the vector’s peritrophic matrix, where the parasites multiply as promastigotes. It is currently unknown whether other developmental forms of *L. tarentolae* exist in the vector and, more specifically, if the metacyclic promastigotes are produced [23]. Infection in lizards can occur through passive cutaneous transmission- via the bite of the vector- or through direct ingestion of the sand fly [24]. In lizard, the parasite lives predominantly as promastigote in the lumen of the cloacae and intestine or into the bloodstream [24]*.* Amastigotes, either free or in monocytes, have been rarely observed in lizards even if both free promastigotes and amastigotes from the blood have been reported [24,25]. The ability of *L. tarentolae* to develop into amastigote forms in lizard is still a matter of debate. While it has been demonstrated that *L. tarentolae* is capable of infecting human phagocytic cells and to differentiate into amastigote-like forms, there is no clear evidence for its efficient replication within macrophages [26,27]*.* Regarding *L. tarentolae* pathogenicity, various studies have shown that some very well-characterized virulence factors, such as GP63, CPB, LPG3 and amastin, present in the pathogenic *Leishmania* (e.g. *Leishmania infantum*, *Leishmania major* and *Leishmania braziliensis*) are expressed also in *L. tarentolae* although with some alterations [4,10,28]. *L. tarentolae* lacks the amastigote specific-A2 gene [29]. A2 gene, which was originally identified in the *Leishmania donovani* complex, is considered one of the main virulence factors in mammals and man. It is supposed to be playing a major role in parasite virulence and visceralization capacity although its exact function has not been fully clarified.

It has been demonstrated that, transfection and expression of A2 in *L. major* transform in a significant way the parasite tropism from cutaneous to visceral infection [30-32]. Similarly, *L. donovani* A2 protein expression in a previous engineered recombinant *Leishmania tarentolae* showed that the parasite’s infectivity and survival in the liver of BALB/c mice is more developed than in the wild-type strain used as control [10]*.* Therefore, it was concluded that, although through an unknown mechanism, the loss of A2 gene was one of the factors, which partly contributed to the loss of virulence of *L. tarentolae* [10].

The recent genome sequencing of *Leishmania tarentolae* Parrot-TarII strain allowed making comparisons among the non-pathogenic protozoan genome and complete genomes of pathogenic *Leishmania* species [4,33,34]. It has been shown that the genomes of the various *Leishmania* species contain a similar number of genes estimated at 8200*.* Despite the 20–100 million years of divergence within the *Leishmania* genus, a comparison of *L. tarentolae* genome with those of the pathogenic *L. major*, *L. infantum* and *L. braziliensis* has shown that there is a strong conservation of gene content and synteny across the genus [4]. A limited number of chromosomal regions diverge between *L. tarentolae* and *L. infantum* while remaining synthenic to *L. major.* Globally, it has been shown that more than 90% *L. tarentolae* genes are shared with the other *Leishmania* species [4]*.*

Despite current knowledge of *L. tarentolae*, as being non-pathogenic to man, here we extend backwards to the Brazilian Colonial Period (end of 18th c/beginning of the 19th c) new evidence for *Leishmania tarentolae* molecular signatures in soft and hard tissue biopsies- bone marrow included- from a male mummy uncovered in Itacambira (Minas Gerais, Brazil). Our finding implies that a systemic spread of the parasite occurred and led us to speculate that a *L. tarentolae* strain, possibly now extinct, had visceralization ability. We also raise a parasitological issue; it is unknown whether any efficient vectors of *L. tarentolae* or susceptible hosts have existed or exist in Brazil today. *De facto*, our findings re-open the discussion concerning the potential survival of an ancient *L. tarentolae* strain within human macrophages and its ability to spread systemically; they also call for further investigations on Brazilian fossil fauna and arthropods in order to shed new light on the host-vector interactions which occurred in the Brazilian semi-arid “cerrado” in its remote and recent past.

## Methods

### Source material

**Figure 1:**
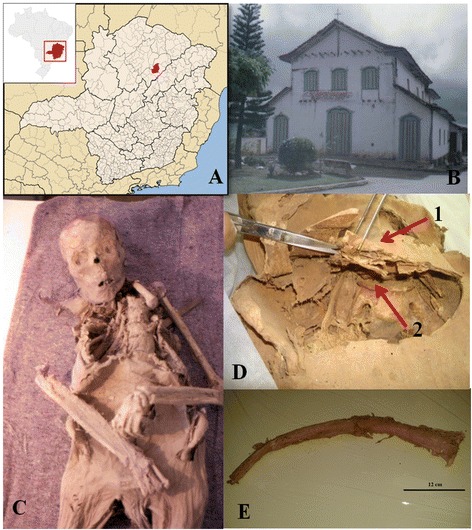
**Municipality of Itacambira, mummy and samples used in the analysis. (A)** Map of the Minas Gerais State, Brazil; the municipality of Itacambira is highlighted in red. **(B)** The church of Sant’Antonio Aparecido in Itacambira. **(C)** Mummy A74. **(D)** Abdominal region of mummy A74 (1. abdominal region and 2. abdominal cavity). **(E)** Rib from mummy A74.

The naturally mummified remains of individual A74 along with other two individuals (A75- an adult male and A78- a six-months-old girl) were unearthed from the topsoil of the church of Sant’Antonio Aparecido in Itacambira (Figure 1A, B).

From the end of the 18th century, a massive colonization occurred in the Minas Gerais region with thousands of Europeans, Africans and South Americans converging in the area. Therefore, the presence of known human *Leishmania* infections (CL, MCL and VL) was investigated in the examined sample.

Conventional PCR was performed on biopsies (1 squared cm each) from the abdominal region, abdominal cavity, cortical bone and bone marrow from the 10th rib of mummy A74. Details of all the biopsies are given in Table 1.

**Table 1:** **Detail of the biopsies taken from mummy A74 and submitted to paleomolecular investigations, the primers used and the results obtained**

**Type of biopsies**	**Primers used**	**Results**
Fragment of tissues adhering to the 10^th^ rib (Figure 1E)	13A: 5′ GGGGAGGGGCGTTCTGCGAA 3′	+
	13B: 5′ SSSCCMCTATWTTACACCAACCCC 3′ [36]	
	13A: 5′ GTGGGGGAGGGGCGTTCT 3′	-
	13B: 5′ ATTTTACACCAACCCCCAG 3′ [37]	
Fragments of tissues from abdominal region (Figure 1D1)	13A: 5′ GGGGAGGGGCGTTCTGCGAA 3′	+
	13B: 5′ SSSCCMCTATWTTACACCAACCCC 3′ [36]	
	13A: 5′ GTGGGGGAGGGGCGTTCT 3′	+
	13B: 5′ ATTTTACACCAACCCCCAG 3′ [37]	
Fragment of tissues from abdominal cavity (Figure 1D2)	13A: 5′ GGGGAGGGGCGTTCTGCGAA 3′	+
	13B: 5′ SSSCCMCTATWTTACACCAACCCC 3′ [36]*	
	13A: 5′ GTGGGGGAGGGGCGTTCT 3′	-
	13B: 5′ ATTTTACACCAACCCCCAG 3′ [37]	
Cortical bone from the 10^th^ rib (Figure 2A)	13A: 5′ GGGGAGGGGCGTTCTGCGAA 3′	**-**
	13B: 5′ SSSCCMCTATWTTACACCAACCCC 3′ [36]**	
Bone marrow from the 10^th^ rib (Figure 2B)	13A: 5′ GGGGAGGGGCGTTCTGCGAA 3′	+
	13B: 5′ SSSCCMCTATWTTACACCAACCCC 3′ [36]**	

* = Sample positive for *Leishmania tarentola*e in the first set of by using Rogers *et al.* [37] primers.

** = Samples from cortical bone and bone marrow analysed in a second round of analyses by using Degrave *et al.* [36] primers. With Degrave *et al.* (1994) primers, a more intense amplification of the PCR products was observed.

### Sample preparation and DNA extraction

ADNA analyses were carried out in ancient DNA dedicated laboratories in the Departamento de Microbiologia e parasitologia, Laboratório de Biologia Molecular de Parasitos, Universidade Federal Fluminense, Niterói, Rio de Janeiro (Brazil).

After arrival in the laboratory, samples were submitted to decontamination procedures consisting of approximately 30 minutes of UV irradiation on each side, manual removal of soft tissue fragments from the bones’ outer surface and a second UV irradiation [35]. The samples were then fine powdered by hand and stored at – 20°C until use.

Extraction blanks, as well as negative PCR controls without template, were included for every DNA extractions and PCR amplification. No positive controls were used. Replicating experiments were performed employing different laboratory space/equipment and enzyme/water aliquots and data were further verified by PCR and sequencing.

The biopsies were incubated in nuclease-free water for 30 minutes at 55°C. Some 200 μl of the resulting solution was used to extract aDNA using the *Purelink Genomik DNA* kit (Invitrogen); the protocol for tissue extraction was applied with the following modifications: incubation in digestion buffer and proteinase k for 2 hours and final elution in 50 μl.

Two pairs of primers directed to the conserved region of the minicircle molecule of the *Leishmania* kinetoplastid mitochondrial DNA were used: 1- 13A(5′-GGGGAGGGGCGTTCTGCGAA-3′) and 13B (5′– SSSCCMCTATWTTACACCAACCCC-3′) [36] and 2-3A(5′– GTGGGGGAGGGGCGTTCT - 3′) and 13B (5′ - ATTTTACACCAACCCCCAG - 3′) [37]. The reactions were performed in a final volume of 50 μl within the same conditions for both primers. The PCR reaction mix contained 5 μl of buffer solution [1X], 1 μL of dNTPs [0,2 μM of each], 2/2 μl the primers set [200 ng], 0,5 μL of Taq Platinum (Invitrogen) [2,5 U] and a total 5uL of extracted DNA (aDNA). Different concentrations of magnesium (Mg) were used depending on the different set of primers: 2 μL of Mg [2 mM] when Rodgers *et al.* [37] primers were used and 1,5 μL of the Mg [1,5 mM] when Degrave *et al*. [36] primers were utilized; the amount of nuclease free water autoclaved of the 32,5 μl for the primer pair second Rodgers *et al*. [37] and 33 μL for the primers pair second Degrave *et al.* [36].

45 cycles of PCR (2720 Thermal Cycler, Applied Biosystems, Darmstadt) with annealing at 50°C for Rodgers *et al.* primers [37] and at 55°C for Degrave *et al*. primers [36] were performed. Amplicons were purified and directly sequenced in an automatic sequencer, 3100 Applied Biosystems, on the platform of Instituto Biomédico da Universidade Federal Fluminense and PDTIS-Fiocruz.

## Results

**Figure 2:**
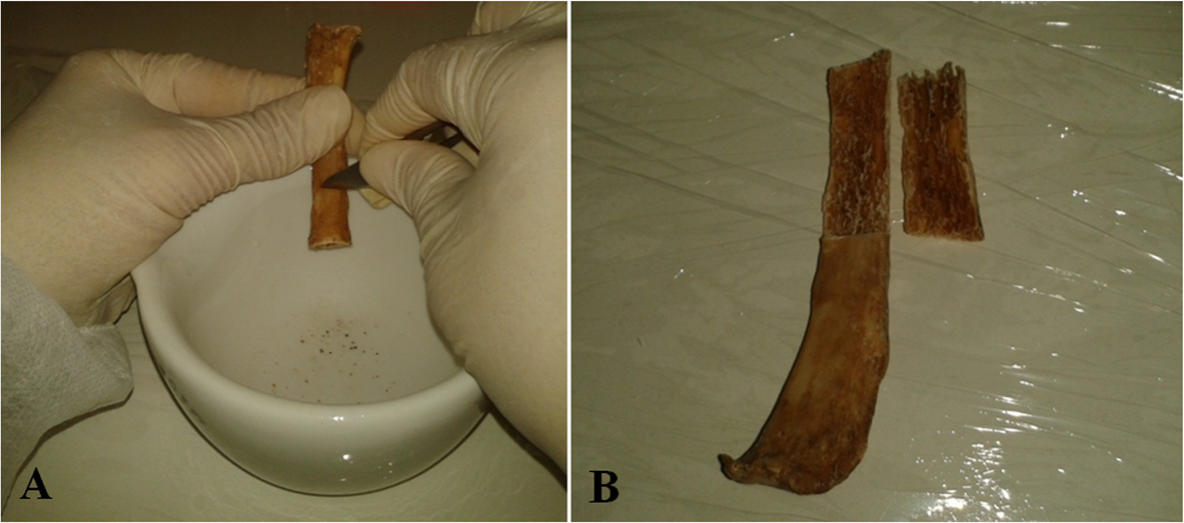
**Samples of rib used in the analysis. (A)** Sampling of cortical bone from the 10th rib. **(B)** Sampling of bone marrow from the 10th rib.

A 120 bp fragment of *Leishmania* spp. kinetoplastid mithocondrial DNA was successfully amplified and sequenced from both bone marrow and cortical portions of rib n°10 (Figure 2A, B) and from soft tissue fragments taken from the skin of abdominal region and cavity (Figure 1D1, D2) belonging to mummy A74.

**Figure 3:**
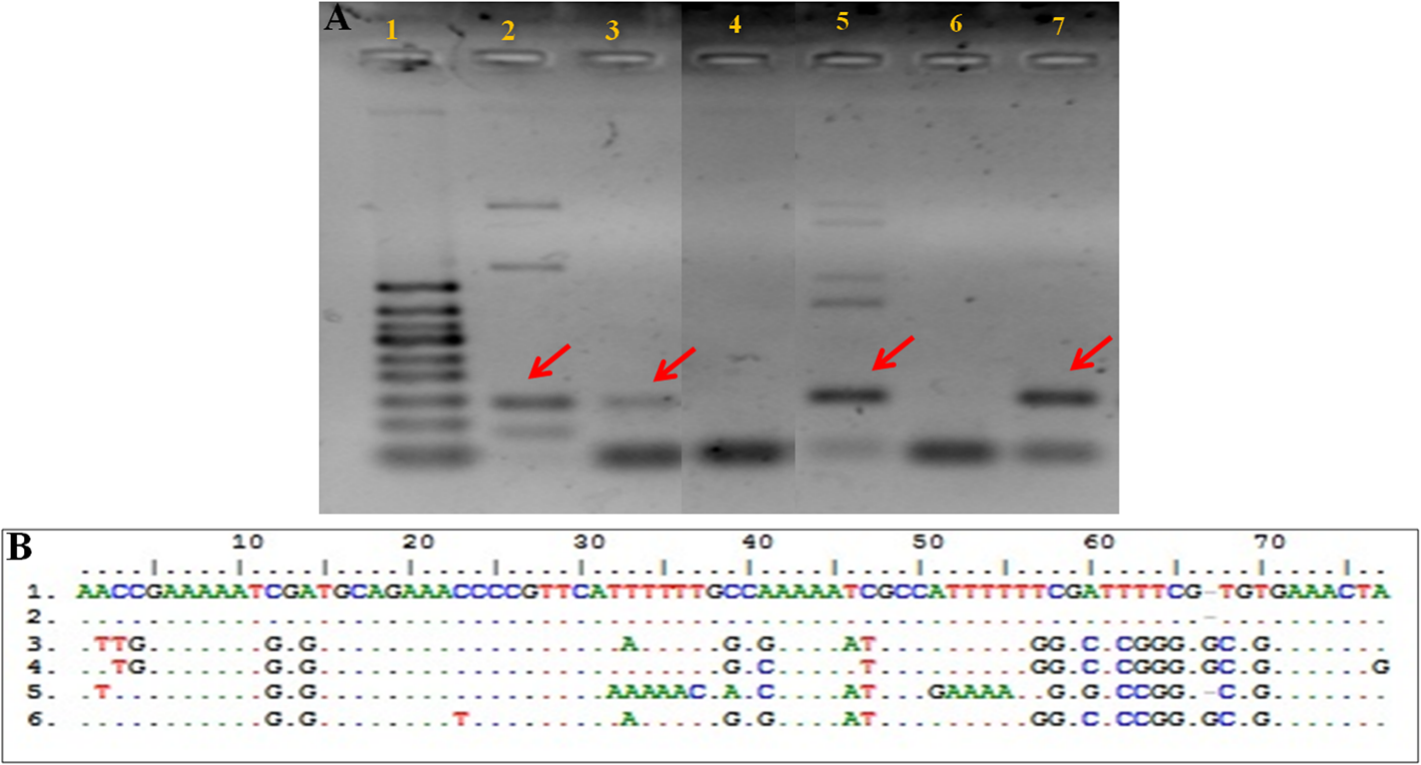
**PCR amplification and alignment for** ***Leishmania*** **spp. in mummy A74. (A)** PCR amplification fragment of a 120 bp fragment of the kinetoplastid mitochondrial DNA of *Leishmania* spp. obtained using Degrave *et al*. (1994) primers [35] (Lane 1: molecular marker 50 bp; lane 2: Tissues from the abdominal cavity; lane 3: Tissues from the abdominal region; lane 4: negative control; lane 5: Tissue adhering to the 10th rib; lane 6: Cortical bone from the 10th rib; lane 7: Bone marrow from the 10th rib). **(B)** Alignment for *Leishmania tarentolae* in mummy A74 (without primers) (1. Reference sequence for *L. tarentolae* from a culture available in *Genbank*: HM579788); 2. *L. tarentolae* aDNA sequence detected in mummy A74; 3. *Leishmania infantum* aDNA from Eleonora di Toledo’s skeletal remains [42] used for comparison; 4. mDNA of *L. infantum* available in *GenBank*: AB678348 used for comparison; 5. DNA of *L. major* available in *GenBank*: EU370908 used for comparison; 6. DNA of *L. donovani* available in *GenBank*: L19877 used for comparison.

The ancient sequence [*Genbank*: KF740726] showed a 100% similarity with those of isolated *L. tarentolae* parasites grown on artificial nutrient media [*GenBank*: HM579788] and 99% similarity with two other sequences obtained from reptilian blood [*GenBank*: X60508, AF380693] (Figure 3A, B). The other *Leishmania* species (*Leishmania donovani, Leishmania major* and *Leishmania infantum*) revealed less probable concordance rates [GenBank: L19877 e-value: 3e-47; EU370908 e-value: 6e-39 and AB678348 e-value: 2e-48]. No amplification was obtained from the blanks.

## Discussion

The small town of Itacambira is located at an altitude of 1,048 masl and has a population of 4,988 distributed in an area of 1,788 km^2^ [38]. The small village of Itacambira was founded in 1674 by mixed Portuguese and native ancestry colonists (the church construction dates back to the end of the 17th century). Its dimensions grew only a century later (18th century) when, due to the extensive exploitation of gold and diamond mines, the first demographic boom occurred and the town’s population finally consolidated (Figure 1A).

Infections with different species of *Leishmania* pathogenic to humans*- L. donovani*, *L. infantum* and *L. braziliensis* are known to have affected individuals from Ancient Egypt [39-42], Renaissance Europe [43,44] and northern Chile [45]. More specifically, it was shown that four South-American women, whose skulls were uncovered in the Atacama Desert and dated to 1000 AD, were infected by *Leishmania braziliensis*. These data showed to be particularly interesting since Chile was and is the only South-American region free from endemic leishmaniases. It was hypothesized that the infection had been introduced from the surrounding countries, possibly Bolivia, through trade routes and migrations [45].

Cutaneous leishmaniasis (CL) and mucocutaenous leishmaniasis (MCL) are supposed to have been endemic to Brazil since ancient times whereas many controversies have been raised on the indigenous origins of VL. Recent studies supported the hypothesis that VL was imported from the Spaniard, via their infected dogs, during the colonization process and in further successive waves [46].

The original host of *L. tarentolae*, the lizard *Tarentolae mauritanica*, was first accidentally introduced, − through marine trades of cork- to Argentina since 1963 from the Mediterranean Basin, gecko’s natural habitat [47].

Neither the presence of the host nor the presence of the parasite has been reported in Brazil so far. Conversely, various other species belonging to the *Gekkonidae* family such as *Hemidactylus agrius* Vanzolini, [48]; *Hemidactylus brasilianus* (Amaral [49]); *Hemidactylus mabouia* (Moreau de Jonnès) [50]); *Hemidactylus palaichthus* Kluge [51]; *Lygodactylus klugei* (Smith, Martin & Swain [52]), and *Lygodactylus wetzeli* (Smith, Martin & Swain [52]) are widely distributed in Brazil.

Numerous experiments were challenged to determine whether New World lizards were susceptible to *L. tarentolae* parasitism*.* Experimental infection challenged against lizards belonging to the *Teiidae* family (e.g. *Cnemidophorus sexlineatus* and *Ameiva quadrilineata*) and to the *Iguanidae* family (*Anolis carolinensis*, *Dipsosaurus dorsalis*, and *Basiliscus vittatus*) [53] showed that only one species, *A. carolinensis*, developed an infection by *L. tarentolae*; the infection was active for 12 hours and, then, cleared.

Two more Brazilian species of lizards belonging to *Gekkonidae* family, *Gonatodes fuscus* and *Hemidactylus turcicus*, were tested for the presence of the wild-type *L. tarentolae* in their bloodstream but did not show any kind of infection [53].

Despite current knowledge, which implies that modern *L. tarentolae* is non-pathogenic to humans, the identification of a 120-bp fragment of the lizard parasite aDNA in soft and hard tissues from individual A74 provides evidence for a successful replication and spread of the parasite in a warm-blooded organism and for a visceralization capacity. We are unable to ascertain whether *L. tarentolae* dissemination to internal organs was completely asymptomatic or not. Apparently no skin lesions were observed. We speculate that species of lizards harboring *L. tarentolae* parasites pathogenic to man might have existed in Brazil in its remote and recent past.

Molecular phylogenetic studies confirm the hypothesis that *Sauroleishmania* species have evolved from the mammalian *Leishmania* [13]. Therefore, it is not unconceivable to hypothesize that *L. tarentolae* maintained its visceralizing capacities also in their new hosts; a subsequent differentiation between strains with ability of infecting mammals and strain with loss of pathogenicity to mammals might have occurred through an adaptation process to the new hosts and vectors. Since the main hosts had become cold-blooded organisms, a selection of strains without pathogenicity to warm-blooded organism might have taken place. While it has been established that the current wild-type *L. tarentolae* strain has lost its patho-antigenicity, it has not been established when, during the microevolution, the gene loss occurred.

On this ground, paleoparasitological investigations have extended our knowledge concerning the potential role played by extinct species and have highlighted the various phases of the parasite-host relation in a given environment, even on a geological scale [54,55].

Following Poinar Jr [54,55], trypanosomids (which include the genus *Leishmania)*, probably existed in the Paleozoic in free-living forms, which were most likely different from the extant species. The discovery of *Paleoleishmania proterus* Poinar & Poinar [56] in the Burmese amber sand fly species *Paleomyia burmitis* Poinar showed that sand flies were already able to transmit trypanosomids to vertebrates in the early Cretaceous, some 100 million years ago (mya).

It was been postulated that free-living trypanosomids were carried from the larval stage, transtadially, into the adult stage and, then, transmitted to vertebrates. The establishment of the parasites in the vertebrate and their subsequent re-acquisition by adult sand flies- a rare event- would have only occurred under ideal conditions but it occurred.

A later discovery in Dominician fossil amber allowed tracing back the history of disease-vectors associations, as microorganisms were preserved *in situ* in the alimentary tract and body cavity of blood sucking insects. A new species of phlebotomine sand fly, *Lutzomyia adketis* sp. n., which was identified in a fossil Dominician amber, was shown to be the vector of *Paleoleishmania neotropicum* sp.n. (Kinetoplastida: *Trypanosomidae)*. The fossil sand fly, *Lutzomyia adketis*, showed to be different from all known extinct and extant members of the genus *Lutzomyia.* The trypanosomid, *Paleoleishmania neotropicum* sp.n., was characterized by the structure of its promastigotes, amastigotes and paramastigotes whereas the vertebrate host of *L. adiketis* remained unknown. Those findings provided first fossil evidence that Neotropical sand flies were vectors of *Trypanosomidae* in the mid-Tertiary (20–30 mya).

It is still unknown whether *Leishmania* originated in the Old or in the New World [55]. Extant species of *Lutzomyia* are restricted to the New World and their host range is quite extensive and includes mammals, birds, reptiles and amphibians [57]. Several extant members of the genus *Lutzomyia* feed on humans and are proven vectors of *Leishmania infantum*, the casual agent of American visceral leishmaniasis. We cannot, therefore, exclude than some extinct species of *Lutzomyia* had acted also as vectors of *L. tarentolae* pathogenic and non-pathogenic strains.

The discovery of a new extinct *Leishmania* species contained in the gut of their vectors embedded in fossilized amber and our recent finding of an ancient strain of *L. tarentolae* in the internal organs of a 300yo individual show the potential of paleoparasitology for the evolutionary studies on parasite-host relations and open new perspectives for future research.

These data emphasize how the phenomenon of parasitism is strictly dependent on three variables: parasite, host and environment [58]. Disease is determined by changes in one or more of the above three system components [59,60]. Alterations of any component of the system occur constantly and, therefore, according to natural selection, changes in parasitism constantly occur; the same is for diseases expression.

## Conclusion

In conclusion, it cannot be excluded that lizards harboring *L. tarentolae* parasites pathogenic to humans might have existed in Brazil in the remote and recent past. It has been demonstrated that *L. tarentolae* parasites are able to invade human macrophages and transform into amastigotes although their replication abilities have never been proved. In this respect, the fact that the parasites might cause asymptomatic infections in humans, cannot be completely ruled out.

In lizards, the parasites are found as free-form bodies. Whole-genome sequencing of *L. tarentolae* [3] showed that more than 90% of the genes are identical to those of the human pathogenic species. However, about 250 genes were absent in *L. tarentolae*, which are precisely the genes specifically expressed in the amastigote stage.

Parasitism may have resulted either by direct inoculation of the parasite through the bite of a sand fly (although no reservoirs or hosts for this pathogen have ever been identified in Brazil) or from the ingestion of raw lizards, which were consumed during periods of food shortage. Ingestion of live geckos has been reported in some semiarid areas of North-eastern Brazil, especially after prolonged droughts, when the population is forced to seek alternative food sources [61]. Itacambira is located in northern Minas Gerais State, a region of transition between the semiarid and the *cerrado*, with a short humid summer and long dry periods during the cold season. As a consequence of these environmental conditions, human populations always suffered water shortage and droughts from late October until the rainy season (April). Therefore, *L. tarentolae* might have been introduced by oral passive transmission through the ingestion of an infected lizard.

A third possible scenario can be foreseen. Since infections caused by pathogenic species of *Leishmania* may remain asymptomatic with persisting parasites circulating in the bloodstream for many years, it cannot be ruled out that *L. tarentolae* might have been imported during the Colonial period from a European colonist or an African slave affected by an asymptomatic infection.

Although modern day *L. tarentolae* wild-type does not produce active infection in humans, *Leishmania tarentolae* microevolution has not been so far elucidated to rule completely out the possibility that some strains might have been pathogenic to humans under certain environmental circumstances.
